# Implementing the WHO Labour Care Guide to reduce the use of Caesarean section in four hospitals in India: protocol and statistical analysis plan for a pragmatic, stepped-wedge, cluster-randomized pilot trial

**DOI:** 10.1186/s12978-022-01525-4

**Published:** 2023-01-20

**Authors:** Joshua P. Vogel, Veronica Pingray, Fernando Althabe, Luz Gibbons, Mabel Berrueta, Yeshita Pujar, Manjunath Somannavar, Sunil S. Vernekar, Alvaro Ciganda, Rocio Rodriguez, Saraswati A. Welling, Amit Revankar, Savitri Bendigeri, Jayashree Ashok Kumar, Shruti Bhavi Patil, Aravind Karinagannanavar, Raveendra R. Anteen, M. R. Pavithra, Shukla Shetty, B. Latha, H. M. Megha, Suman S. Gaddi, Shaila Chikkagowdra, Bellara Raghavendra, Elizabeth Armari, Nick Scott, Katherine Eddy, Caroline S. E. Homer, Shivaprasad S. Goudar

**Affiliations:** 1 Maternal, Child and Adolescent Health Program, Burnet Institute, Melbourne, VIC Australia; 2 Instituto de Efectividad Clínica y Sanitaria (IECS-CONICET), Buenos Aires, Argentina; 3 Women’s and Children’s Health Research Unit, Jawaharlal Nehru Medical College, KLE Academy of Higher Education and Research, Belgaum, Karnataka India; 4 Gadag Institute of Medical Sciences, Gadag, Karnataka India; 5 General Hospital, Gokak, Belgaum, Karnataka India; 6 JJM Medical College, Davangere, Karnataka India; 7 Vijayanagar Institute of Medical Sciences (VIMS), Ballari, Karnataka India

**Keywords:** Caesarean section, Intrapartum care, Labour Care Guide, Partograph

## Abstract

**Background:**

The World Health Organization (WHO) Labour Care Guide (LCG) is a paper-based labour monitoring tool designed to facilitate the implementation of WHO’s latest guidelines for effective, respectful care during labour and childbirth. Implementing the LCG into routine intrapartum care requires a strategy that improves healthcare provider practices during labour and childbirth. Such a strategy might optimize the use of Caesarean section (CS), along with potential benefits on the use of other obstetric interventions, maternal and perinatal health outcomes, and women’s experience of care. However, the effects of a strategy to implement the LCG have not been evaluated in a randomised trial. This study aims to: (1) develop and optimise a strategy for implementing the LCG (formative phase); and (2) To evaluate the implementation of the LCG strategy compared with usual care (trial phase).

**Methods:**

In the formative phase, we will co-design the LCG strategy with key stakeholders informed by facility assessments and provider surveys, which will be field tested in one hospital. The LCG strategy includes a LCG training program, ongoing supportive supervision from senior clinical staff, and audit and feedback using the Robson Classification. We will then conduct a stepped-wedge, cluster-randomized pilot trial in four public hospitals in India, to evaluate the effect of the LCG strategy intervention compared to usual care (simplified WHO partograph). The primary outcome is the CS rate in nulliparous women with singleton, term, cephalic pregnancies in spontaneous labour (Robson Group 1). Secondary outcomes include clinical and process of care outcomes, as well as women’s experience of care outcomes. We will also conduct a process evaluation during the trial, using standardized facility assessments, in-depth interviews and surveys with providers, audits of completed LCGs, labour ward observations and document reviews. An economic evaluation will consider implementation costs and cost-effectiveness.

**Discussion:**

Findings of this trial will guide clinicians, administrators and policymakers on how to effectively implement the LCG, and what (if any) effects the LCG strategy has on process of care, health and experience outcomes. The trial findings will inform the rollout of LCG internationally.

*Trial registration:* CTRI/2021/01/030695 (Protocol version 1.4, 25 April 2022).

**Supplementary Information:**

The online version contains supplementary material available at 10.1186/s12978-022-01525-4.

## Plain language summary

The new WHO Labour Care Guide (LCG) is an innovative partograph that emphasises women-centred, evidence-based care during labour and childbirth. Together with clinicians working at four hospitals in India, we will develop and test a strategy to implement the LCG into routine care in labour wards of these hospitals. We will use a randomised trial design where this LCG strategy is introduced sequentially in each of the four hospitals, in a random order. We will collect data on all women giving birth and their newborns during this period and analyse whether the LCG strategy has any effects on the use of Caesarean section, women’s and newborn’s health outcomes, and women’s experiences during labour and childbirth. While the trial is being conducted, we will also collect qualitative and quantitative data from doctors, nurses and midwives working in these hospitals, to understand their perspectives and experiences of using the LCG in their day-to-day work. In addition, we will collect economic data to understand how much the LCG strategy costs, and how much money it might save if it is effective. Through this study, our international collaboration will generate critical evidence and innovative tools to support implementation of the LCG in other countries.

**Supplementary Information:**

The online version contains supplementary material available at 10.1186/s12978-022-01525-4.

## Background

In the past two decades, considerable efforts have been made to encourage and support pregnant women to give birth in health facilities, where they would, ideally, receive good-quality intrapartum care from skilled health personnel. This has translated into large increases in the global coverage of births attended by skilled health personnel—from 69% in 2006–2012 to 81% in 2013–2018 [1]. However, in many settings (particularly limited-resource settings) women continue to give birth in facilities while denied the option of having a labour companion present, without access to adequate pain relief, and not encouraged to mobilize during labour or adopt a birth position of choice [2, 3]. Interventions such as early amniotomy, oxytocin for augmentation and continuous fetal monitoring are often routinely overused.[2] This overmedicalized and often disrespectful care has helped drive rising Caesarean section (CS) rates and poorer birth experiences for women worldwide [3–5].

An essential component of good-quality intrapartum care is ensuring that women are adequately monitored during labour, such as by prospectively completing a partograph based on regular clinical assessments [6]. In February 2018, WHO published new recommendations on intrapartum care for a positive childbirth experience, including 56 evidence-based recommendations for care of women during labour, birth and the immediate postpartum [7]. The recommendations included updated definitions and durations for first and second stage of labour, based on evidence from systematic reviews [7–9]. The WHO guideline panel concluded that the duration of the first and second stage of labour is highly variable between women, and a cervical dilation rate of 1 cm per hour in the first stage is unrealistically fast for some women. They also concluded that a cervical dilatation rate slower than 1 cm per hour was by itself a poor predictor of adverse birth outcomes and should not be the sole indication for obstetric intervention. These updated recommendations mean that older partograph designs (particularly those with ‘alert’ and ‘action’ lines) were no longer scientifically valid—including the previous simplified WHO partograph. Furthermore, many partograph designs in current use do not monitor the use of supportive care interventions that are known to improve health and experience outcomes, such as labour companionship, women’s mobility, birth position or use of pain relief.

## The WHO Labour Care Guide

To help maternity care providers implement these recommendations, WHO developed a “next-generation” partograph known as the WHO Labour Care Guide (LCG), as well as a LCG user’s manual [10]. The LCG is an innovative labour monitoring-to-decision tool that supports providers in effectively monitoring maternal and fetal status and progress of labour, and offers timely reminders on appropriate clinical and supportive care. The LCG aims to promote women-centred care, stimulate providers to think critically around labour decision-making, and individualise labour monitoring. The LCG was developed through several expert consultations, iterative prototype development and testing, an international survey of maternity care providers, and qualitative research with midwives from six African countries. Following its publication in December 2020, a six-country evaluation project explored the LCG’s usability, feasibility and acceptability in different settings, as well as barriers and facilitators to its use [11, 12]. Using the LCG for managing labour and birth is now the WHO recommended standard for providing intrapartum care internationally [7].

The LCG has been designed for use by all cadres of skilled birth attendants that provide care for women and their babies during labour and birth. It includes assessments and observations that are essential for the care of all pregnant women, regardless of their risk status or where they give birth (i.e., high-resourced or limited-resourced facility). Embedded in the LCG are parameters and alerts to facilitate the use of interventions known to minimise the need for CS, as well as reduce the use of unnecessary augmentation of labour and improve birth experiences. While the LCG was primarily designed to be used for the care of apparently healthy pregnant women and their babies (i.e. women with low risk pregnancies), women at high risk of developing labour complications can still benefit from the LCG as a monitoring tool [20].

Using the LCG might minimize over-diagnosis and under-diagnosis of abnormal labour events, and thus reduce unnecessary use of intrapartum interventions (including intrapartum CS). Furthermore, training in LCG use could possibly discourage the use of ineffective and harmful interventions which WHO recommends against, such as routine shaving, use of enemas, vaginal cleansing, routine amniotomy and routine episiotomy. To achieve any benefits, the LCG would need to be used routinely and effectively during labour and childbirth by providers working in labour wards, typically nurses, midwives or doctors. Our collaboration’s previous work developing and evaluating usability of the LCG in six countries, plus the accumulated experience in the use of previous partographs [13, 14], have identified several barriers to effective labour monitoring using partographs. Hence an implementation strategy that addresses these barriers is required.

**Fig. 1:**
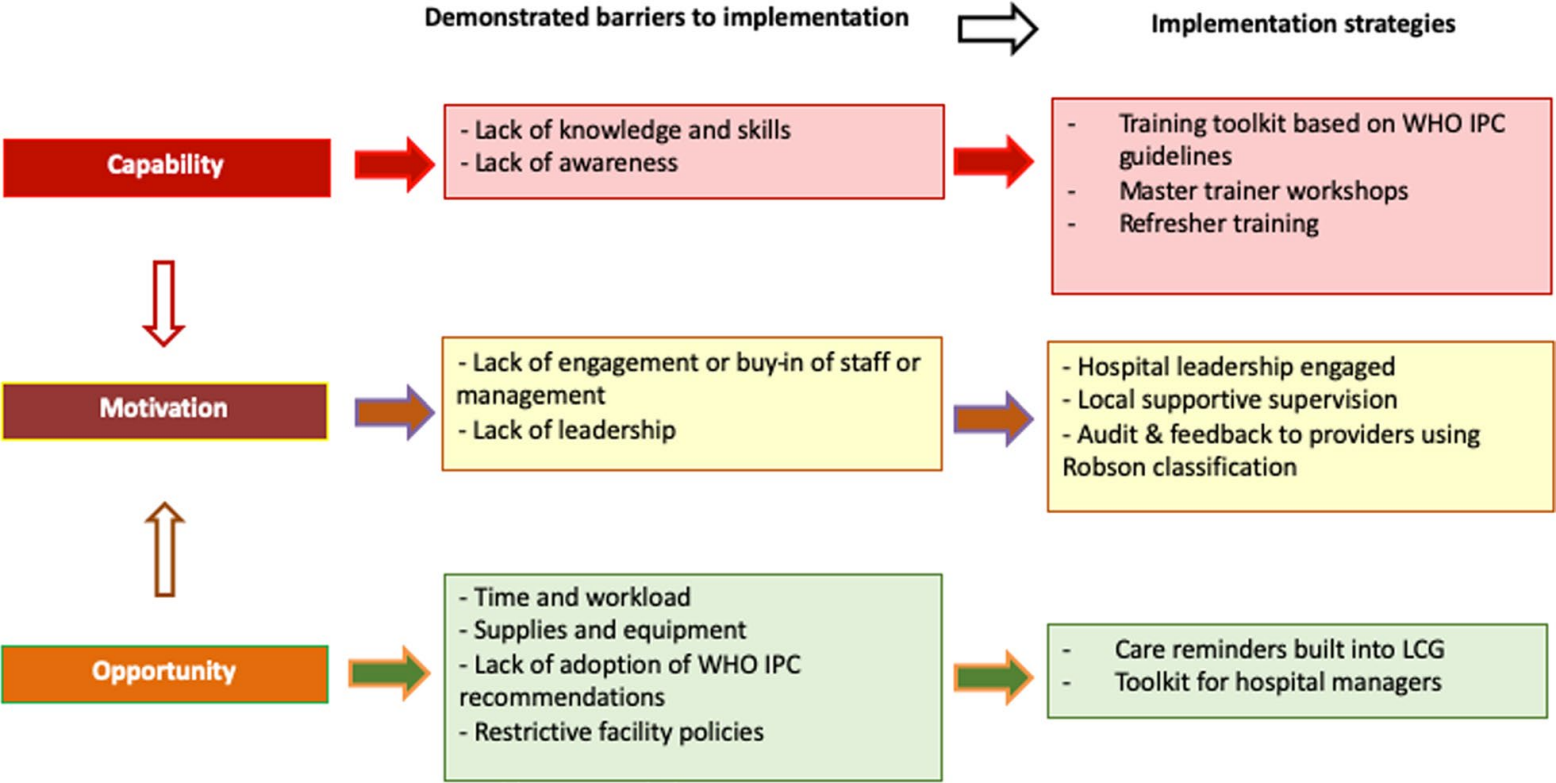
COM-B framework

The COM-B model of behaviour change recognises that individuals must have Capability, Motivation, and both physical and social Opportunity to perform behaviours of interest (Fig. 1) [15]. A number of barriers to routine partograph use have been described in the literature, including a lack of provider knowledge and skills, time limitations, heavy workloads, inadequate equipment and supplies, and restrictive hospital policies [13]. Enabling factors identified include supportive and practical training, regular feedback, teamwork and ensuring staff on labour ward have access to the necessary resources and supplies to monitor and manage women in labour [12]. Implementing the LCG into routine care requires a strategy that can effectively improve healthcare provider’s intrapartum care practices in the context of these barriers and enablers. Such a strategy could optimize the use of Caesarean section, along with other intrapartum interventions, resulting in improved health and experience outcomes for women and babies. However, the effects of such a strategy have not been evaluated in a randomized trial.

## Methods

### Aims and hypothesis

The aims of this study are:

1. Develop and optimise a strategy for implementing the LCG (formative phase)
2. To evaluate the implementation of the LCG strategy compared with usual care (trial phase)

We hypothesize that adoption of the LCG as a labour monitoring-to-decision tool will enhance the quality of care during labour and birth, reduce use of unnecessary interventions and improve support to women giving birth—this might, collectively, reduce intrapartum CS use.

### Overview of study design

**Fig. 2:**
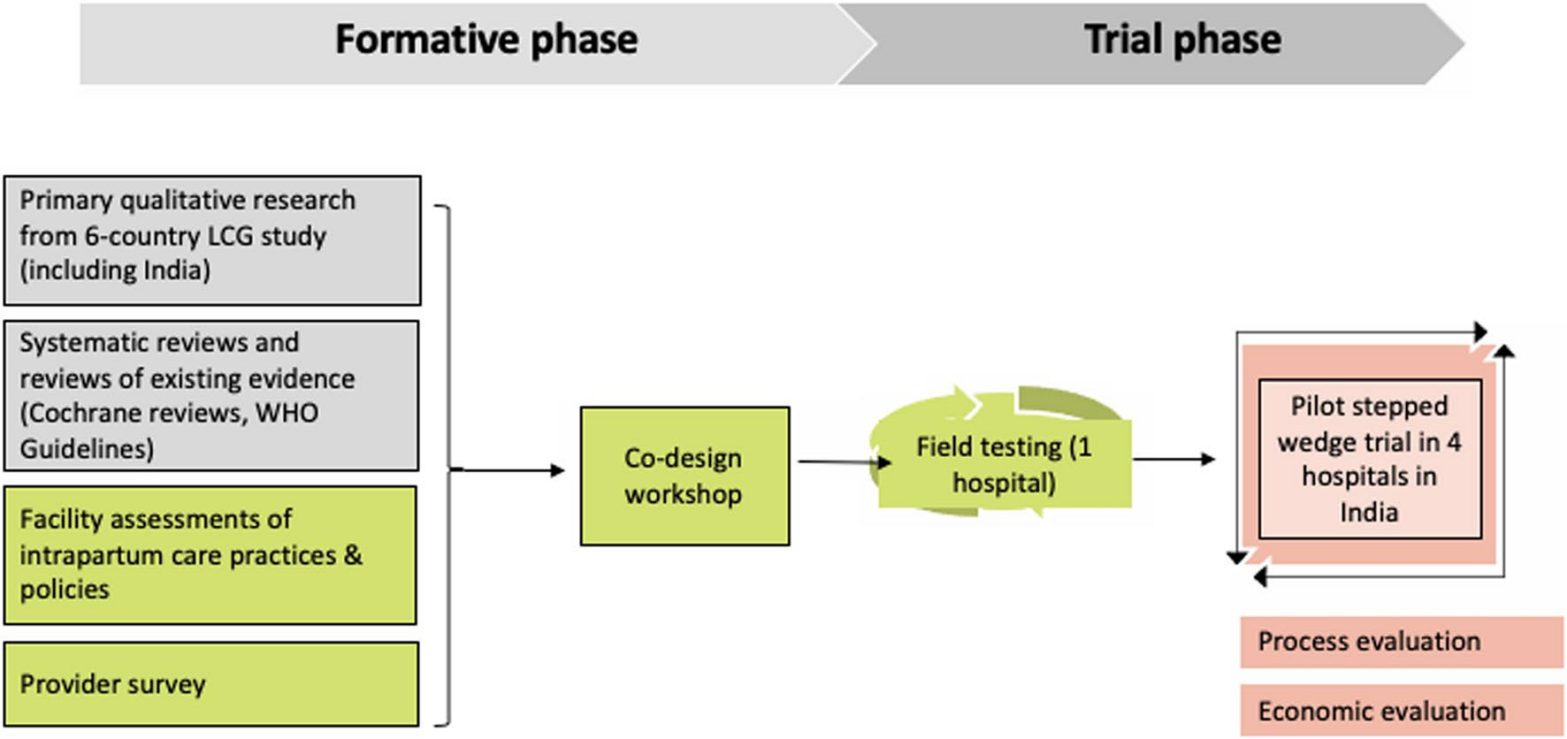
Overview of study activities

This is a two-phased study, comprising a formative phase (facility assessments, provider survey co-design workshop and field testing) and a trial phase (Fig. 2). The trial will be a pragmatic, stepped-wedge, cluster-randomized pilot trial to evaluate the effects of the LCG strategy (intervention). The strategy will target the use of the LCG by maternity care providers through education and training, supportive supervision, and audit and feedback using the Robson Classification. This protocol was developed in accordance with SPIRIT guidance for randomised trial protocols, and the CONSORT statement for stepped-wedge cluster-randomised trials [16, 17]. Burnet Institute is the trial sponsor.

### Study setting

Participating sites are four public hospitals in Karnataka State, India that have 4000 + births annually, capacity to provide comprehensive emergency obstetric care, and are willing and able to participate in a stepped-wedge trial using the LCG, including the implementation of supportive care measures. These hospitals have all either completed or are undergoing the Government of India’s Labour Room Quality Initiative accreditation (LaQshya—a national initiative [18]).

### Formative phase

#### Objectives of formative phase

The specific objectives of the formative phase are: (1) to understand current intrapartum care policies and practices in the study hospitals; (2) to identify potential factors that may affect adoption of the LCG strategy in study hospitals; and (3) to optimise relevant intrapartum care policies and practices in study hospitals prior to trial commencement.

During an in-person visit to each participating hospital by investigators, a pre-designed facility assessment form will be completed**.** This form will collect information on the current intrapartum care environment, policies, and practices at each hospital. The assessment is informed by the requirements described in the WHO 2018 intrapartum care recommendations, WHO 2018 quality standards for maternal and newborn care, and a 2019 systematic review of maternity facility assessment tools [7, 19, 20]. It uses a mix of open and closed-questions, with information collected through consultation with senior hospital staff and direct observations. The facility assessment covers: provision of basic and comprehensive emergency obstetric services; facility services; current workforce distribution; referral processes; audit procedures; physical resources; availability of medicines, working equipment and supplies; current intrapartum care policies and clinical protocols (particularly respectful labour and childbirth care policies (labour companionship, effective communication, pain relief, oral fluid and food intake, mobility in labour and birth position of choice, continuity of care); current training and education provided to staff; accreditation status and current labour ward monitoring and documentation practices. A paper-based survey of maternity care providers working in labour ward (obstetricians, residents, midwives or nurses) will also be conducted at all four hospitals. They will be invited to provide informed consent and complete the survey anonymously, which will include questions on: sociodemographic and professional information; how labour and childbirth care is currently conducted at their hospital; factors influencing intrapartum care at their hospital; current education and training activities at their hospital and factors potentially influencing the LCG strategy. Response options include a combination of dichotomous, Likert scales, short answer response and multi-option formats.

Refinement of the LCG strategy involves triangulation of findings from the formative phase by the research team. Specifically, we will tabulate formative findings to identify barriers and enablers to the LCG strategy, with main findings and themes identified. It is also anticipated that during the formative phase we will identify those hospital policies that will need to be revised or updated before the LCG strategy can be introduced. For example, supportive care interventions such as encouraging mobility during labour, or offering a labour companion of choice to be present during labour and birth may not be part of routine care in participating hospitals.

A co-design stakeholder workshop will be held over two days via Zoom. Workshop participants will include maternity care providers from participating hospitals, as well as the international study team. On day 1, the study team will present the formative findings and the proposed components of the LCG strategy. On day 2, facilitated group discussions will be held on how best to implement the LCG in participating hospitals. Outputs of this workshop will be used to report the intervention development process; and to finalise the manual of operations and LCG training materials prior to the trial phase. We will also conduct field-testing of the LCG strategy and study materials in a separate non-study hospital, prior to commencement of the trial phase.

### Trial phase

#### Objectives of trial phase

Primary objective:

1. Evaluate the effect of implementing the LCG strategy on CS rate amongst women in Robson Group 1
  Secondary objectives:
2. Evaluate the effect of implementing the LCG strategy on women’s health and process of care outcomes, and women’s experiences of care
3. Conduct a process evaluation and economic evaluation on the implementation of the LCG strategy

#### Study design

In this phase we will conduct a stepped-wedge, cluster-randomised pilot trial at four hospitals. The LCG strategy includes the use of training, supportive supervision, and audit and feedback, all of which are directed at the cluster (hospital) level, with the intention of improving routine labour and childbirth care. It aims to increase compliance with the current WHO recommendations for intrapartum care. The cluster design has been adopted as it would not be possible nor practical to individually randomise women to LCG versus usual care—the likelihood of cross-contamination would be very high. A stepped-wedge approach is necessary as the LCG is WHO’s standard tool aligned with their latest intrapartum care recommendations [7], and it would not be feasible to use a parallel-group design in this context. It will also prevent disappointment bias from hospitals dropping out if they are not randomised to the intervention. Hospitals are the randomization unit (i.e., clusters). The target population of the LCG strategy are maternity care providers (doctors, midwives or nurses) who are working in labour wards at participating hospitals during the study period and are expected to use the LCG as part of their routine clinical responsibilities. The trial will be conducted in the same four hospitals that participated in the formative phase.

#### Randomisation

**Fig. 3:**
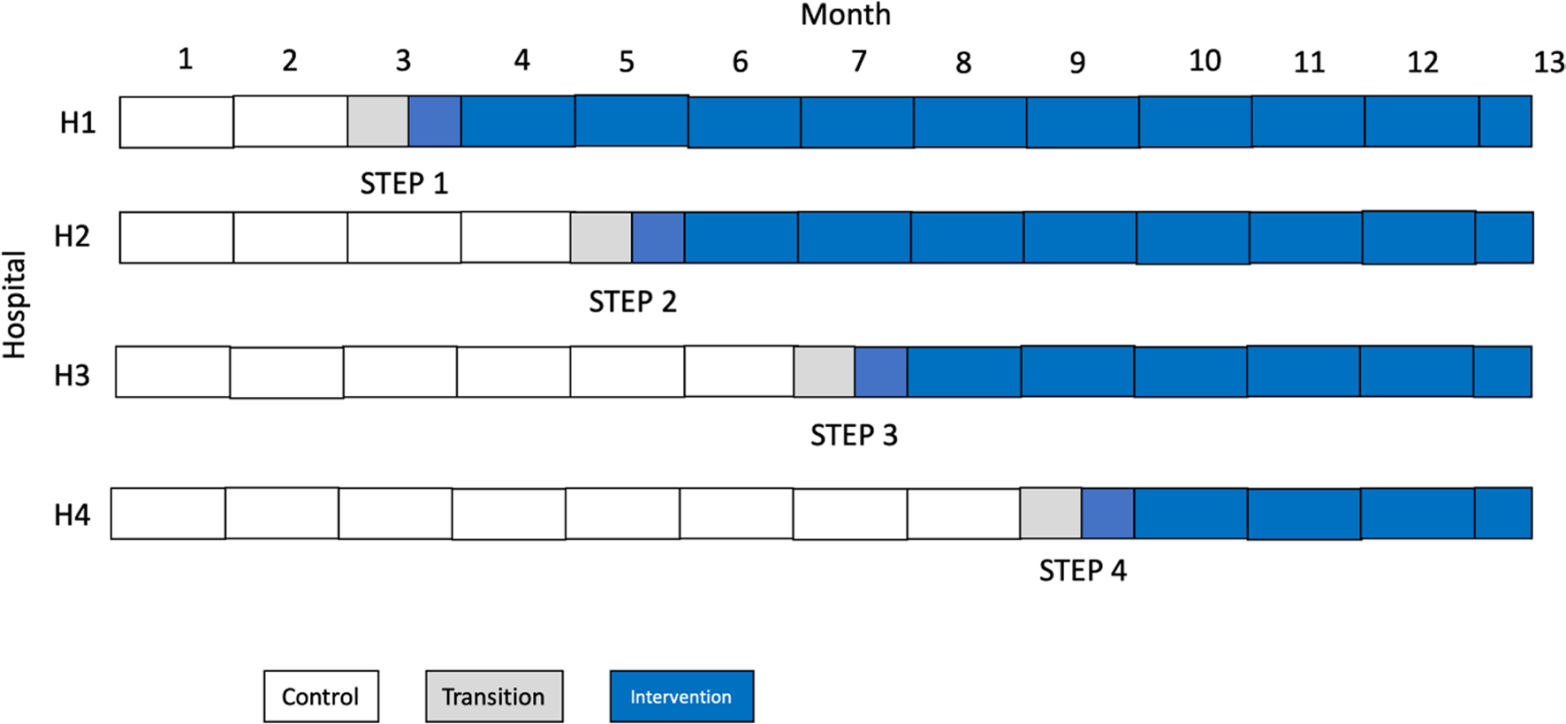
Stepped wedge cluster randomisation process

All four hospitals will initially be observed under usual care for two months (Fig. 3), with the intervention rolled out sequentially (in random order), with a hospital transitioning to the intervention every 2 months at each step (i.e. four sequences). A half-month transition period is included for each hospital to allow for the intervention to be fully adopted. Prior to trial commencement, hospitals will be randomly assigned to one of the four sequences (H1, H2, H3, or H4) for time of crossover from control condition to intervention condition, using a computer-generated list of random numbers (generated by the study statistician). Investigators and hospital teams will be blinded to this allocation sequence, with only the next hospital being revealed at each timepoint. The allocation is revealed 1 month prior to the randomisation, to allow time for scheduling LCG training activities. As the trial is evaluating a complex intervention using a stepped wedge design, blinding of hospital staff, individual women, research staff and statistician is not possible.

#### Participants

This trial will evaluate a complex intervention that primarily targets healthcare providers. They will be trained to use the LCG, which has been designed to improve the care of women and their babies during labour and birth. Using LCG includes assessments and observations that are essential for the care of all pregnant women, regardless of their risk status. The LCG was primarily designed to be used for the care of apparently healthy pregnant women and their babies (i.e. women with low risk pregnancies). Women at high risk of developing labour complications may require additional monitoring and interventions or individualized labour and childbirth care in accordance with local standards, though they will still be monitored with the LCG [21]. Obstetric care providers at these hospitals will also participate in audit and feedback activities. While the LCG strategy does not directly interact with women giving birth, it is expected to improve the care provided to women.

#### Control and intervention

During the control period, each hospital will provide labour care according to their standard practice, which is using the WHO simplified partograph for routine labour management. Prior to commencement of the trial, all hospitals and labour ward providers will undergo a brief standardised training on correct use of the WHO simplified partograph.

The trial intervention has two main components:

*Part A—implementing the LCG:* The LCG will be used by maternity care providers to monitor women during labour and childbirth and help promote best-practice intrapartum care. Providers will undergo an initial standardised two-day training workshop using the WHO LCG Manual and co-designed training package on how to use the LCG. The training will be provided by senior obstetricians at the participating hospitals who have previously completed a “training of trainers” workshop, with support from a LCG master trainer. Weekly case-based learning using LCG for labour ward providers will continue for an 8-week period after the initial training workshop. This case-based learning program is based on the “low-dose, high-frequency” approach that has proved effective in recent maternal and newborn health education interventions [22]. Periodic refresher training for existing staff and additional training for new staff will also be conducted to ensure that all providers working in labour ward have received adequate LCG training.

In addition, the introduction and routine use of LCG will be communicated to staff through posters, checklists in medical records, and other staff awareness and engagement activities. Blank LCGs will be made widely available in labour ward, and all copies of simplified WHO partographs will be removed at time of randomization. Supportive supervision of LCG use by providers will also be employed by senior clinical staff in labour wards. By supportive supervision, we mean that clinical supervisors will regularly observe labour ward staff completing the LCG in real time, provide constructive feedback and support, and ensure errors are corrected and queries resolved as they arise. This approach aims to encourage open, two-way communication, facilitate problem-solving, and provide regular follow-up and review with staff by supervisors to ensure that appropriate intrapartum decision-making is being applied.

*Part B—audit and feedback using Robson Classification*: Audit and feedback is a widely used strategy to promote evidence-based practice, where clinical performance indicators are provided to healthcare providers to drive improvements. A 2012 Cochrane review of 140 studies using audit and feedback interventions concluded that they can improve clinical practice, though effects are often modest [23]. Factors associated with improved outcomes include: when the source of feedback is a supervisor, colleague or respected opinion leader; delivered at least monthly; provided on more than one occasion; is both verbal and written; includes explicit targets and an action plan. In their 2018 guideline, WHO recommends that “implementation of evidence-based clinical practice guidelines, caesarean section audits and timely feedback to health-care professionals are recommended to reduce caesarean births”. This was on the basis of high-certainty evidence that showed that implementation of guidelines combined with audit and feedback could slightly reduce CS rates in women with low-risk pregnancies (− 1.7% risk difference) [24]. WHO also recommends that countries use the Robson Classification for assessing, monitoring and comparing their CS rates over time [25]. The Robson Classification organises all births in a facility into one of 10 mutually exclusive, all-inclusive groups, on the basis of parity, previous CS, onset of labour, fetal presentation, number of neonates and gestational age (term or preterm) [26]. Randomised hospitals will be provided with an intensive, 2-h training workshop on how to interpret and classify CS data by Robson Classification and how to conduct audit and feedback sessions at their hospital sites.

Robson Classification tables will then be prepared by an independent analyst based on data collected during the trial and shared directly with the study hospital on a monthly basis (from time of randomization until end of trial). The trial steering group will be blinded to these reports. Hospital leads will be responsible for organising audit meetings each month, where these Robson Classification tables will be presented to labour ward staff at monthly meetings, with structured discussions on how to improve performance. There will also be opportunities for staff to reflect on their experience with the LCG and identify areas where they feel they need more improvement or support. These meetings will be embedded within usual audit and feedback and clinical meeting activities at the hospital and meeting minutes will be taken by a delegated staff member.

#### Outcomes

The population of interest is all women giving birth from 20 weeks’ gestation onwards in participating facilities during the study period. The primary outcome is the use of CS amongst women in Robson Group 1 (i.e. women who are nulliparous, singleton, cephalic, ≥ 37 weeks’ gestation, presenting in spontaneous labour).

While Robson Group 1 represents a subset of all women giving birth in a hospital (usually 30% or more of all women), it is in this group of largely low-risk women where overuse of CS is often detected. For example, WHO advises that CS rates of 10% or less are achievable in Robson Group 1 with good maternal and perinatal outcomes, on the basis of a reference group of 42,637 women giving birth 66 hospitals across 22 countries [26]. However in some hospitals in low- and middle-income countries (LMICs), CS rates in Robson Group 1 exceed 20% to 25% [27]. As this subgroup is quite large and is composed largely of low-risk women in whom CS is often over-used, it is in this group of women where the benefits of LCG on CS use are most likely to be detected. The LCG strategy is however unlikely to reduce CS rates in higher-risk women, such as those with multiple pregnancies (Group 8), or with an oblique lie (Group 9) which account for only a few percent of all women giving birth and in whom the CS rate is necessarily high. Women who are admitted for antepartum CS do not experience labour, and therefore do not require a LCG (or any partograph) as part of their care. We therefore do not anticipate that antepartum CS use would be directly affected by increasing use of LCG.

Table 1 lists the secondary outcomes that will be used in the trial. These include process of care outcomes related to use of intrapartum interventions, maternal health outcomes, fetal and neonatal outcomes, and women’s experience outcomes. The women’s experience outcomes will be measured through a pre-tested, interviewer-administered survey in a sample of postpartum women, completed prior to discharge from hospital. Considering the large volume of women who will give birth during the study period, we will invite only a subgroup of women to complete the postpartum survey. Eligible women are those in Robson Group 1 or 3, aged >  = 18 with a liveborn baby, and who are willing and medically able to complete a survey. We will approach all such women who were enrolled in the last 15 days of the 2-month baseline period, and the last 15 days of each 2-month step in each cluster. An informed consent process will be conducted by trained research staff, and women will be welcome to consider their participation in this survey freely, with no time restrictions. Interviewers will be trained to follow best practices of interviewing techniques and minimize the introduction of bias.

**Table 1:** Secondary outcomes

Outcome	Outcome definition
Maternal process of care and health outcomes	
CS rate in women in Robson Groups 1 and 3	Numerator: number of women undergoing CS Denominator: number of women in Robson Groups 1 and 3
CS rate in women in Robson Groups 1 to 5	Numerator: number of women undergoing CS Denominator: number of women in Robson Groups 1 to 5
Overall CS rate	Numerator: number of women undergoing CS Denominator: number of women giving birth
Augmentation with oxytocin during labour rate	Numerator: number of women given oxytocin for augmentation during labour Denominator: number of women who experienced spontaneous labour
Artificial rupture of the membranes rate	Numerator: number of women who had artificial rupture of membranes Denominator: number of women who experienced spontaneous labour
Episiotomy rate	Numerator: number of women who had episiotomy Denominator: number of women with vaginal birth
Operative vaginal birth rate	Numerator: number of women who had operative vaginal birth (forceps or vacuum) Denominator: number of women with vaginal birth
Duration of hospital admission	Total length (hours) of hospital admission for childbirth
3^rd^ or 4^th^ degree tears	Numerator: number of women experiencing 3rd or 4th degree tears Denominator: number of women giving birth
PPH requiring uterine balloon tamponade or surgical intervention	Numerator: number of women requiring uterine balloon tamponade OR surgical intervention for PPH Denominator: number of women giving birth
Suspected or confirmed maternal infection requiring therapeutic antibiotics	Numerator: number of women with clinical signs or symptoms of maternal infection AND therapeutic antibiotics were required Denominator: number of women giving birth
Fetal / neonatal outcomes	
Antepartum stillbirth	Numerator: fetal death prior to admission, or after admission but before onset of painful uterine contractions with cervical changes Denominator: all born babies
Intrapartum stillbirth	Numerator: fetal death after admission AND after onset of painful uterine contractions with cervical changes Denominator: all born babies
Apgar score < 7 at 5 min	Numerator: liveborn babies with Apgar < 7 at 5 min Denominator: liveborn babies
Bag and mask ventilation of newborn	Numerator: use of continuous bag and mask ventilation of newborn for > 1 min Denominator: liveborn babies
Mechanical ventilation of newborn	Numerator: use of mechanical ventilation of newborn Denominator: liveborn babies
Prolonged (> 48 h) admission in NICU	Numerator: admission to NICU for > 48 h Denominator: liveborn babies
Newborns requiring NICU admission for hypoxic ischaemic encephalopathy	Numerator: admission to NICU for suspected or confirmed Denominator: liveborn babies
Neonatal death	Numerator: neonatal death in a liveborn infant by day 7 or discharge (whichever came first) Denominator: all liveborn babies
Women’s experience outcomes	
Woman’s experience with labour companion	Numerator: women who reported a labour companion was present during labour or birth Denominator: women in Robson Group 1 or 3 who complete postpartum survey
Woman’s experience of being offered pain relief	Numerator: women who reported that they were asked whether they would like any pain relief Denominator: women in Robson Group 1 or 3 who completed the survey
Women’s satisfaction with their pain management during labour and birth	Numerator: women who reported being very satisfied or somewhat satisfied with how their pain was managed during labour and birth Denominator: women in Robson Group 1 or 3 who completed the survey
Woman’s experience of being encouraged to drink oral fluids	Numerator: women who reported that a health worker encouraged them to drink water Denominator: women in Robson Group 1 or 3 who complete postpartum survey
Woman’s experience of being encouraged to eat food	Numerator: women who reported that a health worker encouraged them to eat food Denominator: Women in Robson Group 1 or 3 who complete postpartum survey
Woman’s experience of mobilising during labour	Numerator: women who reported that a health worker encouraged them to walk around during labour Denominator: women in Robson Group 1 or 3
Woman’s experience of birth position of choice	Numerator: women who reported that a health worker asked them which birth position they preferred Denominator: women in Robson Group 1 or 3 who complete postpartum survey
Woman’s experience of time health worker spent with them	Numerator: women who reported being very satisfied or somewhat satisfied with amount of time health worker spent with them during labour Denominator: women in Robson Group 1 or 3 who complete postpartum survey
Women’s satisfaction with the way health providers communicated with them	Numerator: women who reported being very satisfied or somewhat satisfied with the way health workers communicated with them during labour and birth Denominator: women in Robson Group 1 or 3 who complete postpartum survey
Woman’s experience of privacy	Numerator: number of women who strongly agreed or agreed that their privacy was respected during examinations and treatments Denominator: women in Robson Group 1 or 3 who complete postpartum survey
Women’s experience of being asked permission	Numerator: number of women who said their health worker always asked permission before examinations and treatments Denominator: women in Robson Group 1 or 3 who complete postpartum survey
Woman’s overall experience of care	Numerator: number of women who strongly agreed or agreed that they felt satisfied with their labour and birth experience Denominator: women in Robson Group 1 or 3 who complete postpartum survey

#### Data sources and participant timeline

Non-identifiable data on all women giving birth at 20 weeks’ gestation or more will be collected and entered onto trial data collection forms by trained data collectors using a tablet. Data on maternal characteristics, use of intrapartum interventions, as well as health outcomes for mother and baby will be collected from medical/birth records, with clarification sought from providers if required. The period of interest is the time of admission for childbirth until the time of discharge, transfer, death or until 7 days after admission (whichever comes first). Data on women’s experience outcomes will be measured through an pre-tested interviewer-administered survey.

#### Sample size

This trial will use a health outcome (CS rate in Robson Group 1) to evaluate the effects of the LCG strategy. However, as this is a relatively new and complex intervention, the effect size and intra-cluster correlation (ICC) is difficult to estimate. One important output of this pilot trial will be to better estimate these measures for future, larger trials. The four hospitals collectively have on average approximately 24,000 births per year (around 4000 births every 2 months) and the overall CS rate across all hospitals was approximately 44% in 2020. We estimate the current CS rate in women in Robson Group 1 in these four hospitals to be at least 40%. Across all four hospitals, approximately 1300 women in Robson Group 1 give birth every 2 months (i.e., an average of 325 women per cluster). The trial will use 4 steps, 1 cluster in each step, a CAC of 90% and a cluster size of 300 women per step.

The trial will provide 92% power to detect a 25% reduction in the Robson Group 1 CS rate from 40 to 30% (ICC 0.02). Should the ICC be higher than expected (eg: 0.05), or the CS rate in Robson Group 1 is lower than expected (eg: 30%) we will have > 80% power to detect a 30% relative reduction (Table 2). A separate power estimation was also performed for 240 women per cluster per step—the trial will provide 87% power to detect a 25% reduction in the Robson Group 1 CS rate from 40 to 30%.

**Table 2:** Power estimation considering 4 steps, 1 cluster in each step, a CAC of 90% and a cluster size of 300 women (coefficient of variation of cluster size 0.60)

Baseline CS rate in Robson Group 1	ICC	Relative reduction			
		35%	30%	25%	20%
40%*	0.01	100%	99%	97%	86%
	0.02	100%	99%	92%	76%
	0.05	96%	88%	74%	54%
30%**	0.01	99%	96%	88%	70%
	0.02	97%	91%	78%	58%
	0.05	85%	73%	56%	38%

^*^CS at the intervention period: 26%, 28%, 30%, 32%

^**^CS at the intervention period: 19.5%, 21%, 22.5%, 24%

#### Data collection, management and confidentiality

Trial data will be collected via standardised electronic data capture forms on tablets, according to the trial Manual of Operations. Designated forms will also be available for serious adverse events, protocol deviations or protocol violations. Collected data will be entered into a web-based, Good Clinical Practice (GCP) compliant data management platform (REDCap [28]), overseen by the site data managers. These data will be managed centrally by a trial data management team (IECS, Argentina). A validation system has been built into the data management system to ensure consistency, accuracy and completeness of the data collected. The following measures will be taken to ensure confidentiality:

- Data collection forms and the database will identify facilities, providers and women using unique ID numbers only. Personal or identifying information will not be collected on data collection forms or in the database (i.e. non-identifiable).
- Site logs can contain personal information to link participants to unique IDs. These will be kept separate from the data collection instruments and kept securely with project documents at the participating site.
- All project documents will be kept securely under lock and key in project offices and will not be accessible, other than to the project team.
- Data will be entered by unique ID number (no personal or identifying information) into the password-protected data management systems/computers, to which only project staff will have access.
- Participating hospitals will have access to data for their site only.
- The final report will not contain any personal identifying information (i.e. facilities, providers and women will be identified only through unique codes).

Several measures will be taken to ensure data quality:

- All named investigators and study staff will have up to date GCP certification.
- A Manual of Operations will be developed and shared with all sites to guide and standardize study activities.
- Prior to project commencement, training sessions (based on the Manual of Operations) will be held for project staff, to ensure the study requirements and activities are clear.
- Captured data will be checked by IECS team and any discrepancies or queries will be sent to site study staff members for corrections.
- A validation system will be built into the data management system to ensure consistency, accuracy and completeness of the quantitative data collected (such as range checks, consistency checks and skips).

#### Serious adverse event reporting

The interventions being tested in this trial are the WHO standard of care, hence there are no adverse events which would be anticipated as a unique consequence of the trial. No expedited reporting of non-serious adverse events is proposed. Considering that this trial will be conducted in hospitals in limited-resource settings, we anticipate that maternal, fetal or neonatal death/s will occur during the trial period. Any such death will be routinely recorded as a serious adverse event (SAE). A SAE form will be also routinely completed if any of a pre-specified list of severe maternal or newborn morbidities occurs. Investigators may complete a SAE form for other serious events or conditions, as required.

#### Statistical methods and analysis

The study statistician will be responsible for overseeing data management, as well as the execution of the pre-planned analyses (and any subsequent secondary analyses). A Statistical Analysis Plan has been developed (Additional file 1) which has been reviewed by the Data and Safety Monitoring Committee (DSMC). As evident from the primary and secondary outcomes, Robson Classification tables (including all 10 groups—the size of the population, the CS rate, and the relative and absolute contribution of each group to the overall CS rate) will be prepared as part of reporting of results. An interim analysis will be prepared for the DSMC meeting, approximately half-way through the trial.

All analyses will be by intention-to-treat, with clusters analysed according to their randomised allocation. Maternal baseline characteristics (for example age, parity, and pregnancy type) will be summarised as means and standard deviations, medians and inter-quartile ranges, or numbers and percentages, as appropriate, grouped by trial arm. A trial diagram will be presented showing the outcome rate by cluster and by step. The primary comparison will be composed by the characteristics of the women enrolled at the control period versus those enrolled at the intervention period. For the primary outcome and secondary outcomes, a generalized estimating equation (GEE) will be used to estimate the effect of the intervention with respect to the population average. An exchangeable correlation structure will be assumed and the binomial distribution with log link function will be considered. The relative risk and the 95% confidence interval will be reported as the size effect. The model will be constructed considering two variables: a binary indicator for treatment—indicating whether the observation was made during the control or the intervention period and a categorical variable indicating the step (1–4). A method of bias correction and a degree of freedom approximation will be apply to maintain the validity of the estimations due to the small number of clusters [29]. The Manck and DeRouen correction with N-2 degree of freedom will be used as it is the most conservative option [30]. The same methodology will be used for the outcome “duration of hospital admission in days” changing the distribution into a Poisson as the possible values goes from 0 to 7 + 2.

The analysis will be carried out using R version 4.1.1.

#### Trial monitoring

An initiation site visit was conducted by the site investigators to participating hospitals prior to commencing the formative phase. Prior to trial phase, an additional site visit will be conducted to provide training, ensuring GCP compliance and correct implementation of the project activities, as well as ensure data collection and management procedures are in place. Monitoring of the trial during recruitment will be co-ordinated by the principal investigators. This will be done using online monitoring of data entry and management (via REDCap), as well as regular, in-person, monitoring visits to participating hospitals. These visits will be timed to coincide with project preparation and data collection phases, to evaluate protocol adherence, and verify that the rights and well-being of the trial participants are protected, that all regulatory documents are in place, and data management processes and procedures are performed correctly. Additional visits may be carried out depending on the hospital activity and performance. At each site, a random sample of data collection forms will be checked against medical records or other source documents to verify that the trial records are accurate, complete and verifiable. All monitoring activities will be performed in accordance with a pre-designed standard monitoring procedures checklist. Investigators and staff performing monitoring visits will determine, if necessary, any actions to be taken after each visit. They will discuss challenges and problems encountered at the participating hospital and analyse potential solutions.

The DSMC has been appointed, composed of three qualified professionals who are not involved in the running of the trial. This includes an independent Chair, a statistician and an obstetrician/gynaecologist, all of whom do not have important conflicts of interest. A DSMC Charter was developed by the trial team and approved by the DSMC members. The DSMC will meet at least annually unless there is a specific reason to amend the schedule. Selected maternal and newborn outcomes will be reviewed in aggregate at any DSMC meetings to assess safety (maternal and newborn mortality and severe morbidity outcomes).

#### Process evaluation and economic evaluation

Alongside the trial, we will conduct a process evaluation to assess the extent to which the interventions have been implemented as intended. We will adopt the UK Medical Research Council guidance on process evaluations of complex interventions to evaluate this based on three main domains: context, implementation, and mechanisms of impact [31]. The implementation outcomes of interest are fidelity (was the LCG strategy delivered and engaged with as planned?), dose (how much of the LCG strategy was delivered?), reach (how many healthcare providers used the LCG strategy?) and adaptation (what, if any, modifications were made to the LCG strategy to adapt to the study context, and achieve the study protocol?).

These will be explored through multiple methods, including standardized facility assessments, in-depth interviews and surveys with providers, audits and scoring of a random sample of completed LCGs, direct observations of labour ward environments and clinical staff, and a review of relevant documents (such as LCG training attendance registers, logbooks and meeting minutes). These data collection activities will be conducted 3–6 months after hospital randomisation, and will involve healthcare providers at all participating hospitals, including nurses, midwives, and doctors of different levels of experience and management responsibilities. Qualitative data will be analysed using a combined deductive framework and inductive thematic analysis approach and quantitative findings will be reported descriptively.

We will also conduct an economic evaluation which will focus on the implementation costs of the intervention, cost-effectiveness of the LCG strategy (in terms of cost per CS averted) and a sensitivity analysis to identify key cost and epidemiological inputs that have the greatest influence on total cost and cost-effectiveness. The main costs for the LCG implementation strategy are staff time, hence we will request site investigators to estimate required staff time/costs for all intervention-related activities. We will also collect data related to staff time, supplies, equipment and medicines required for all possible modes of birth (spontaneous vaginal birth, operative vaginal birth, antepartum Caesarean section or intrapartum Caesarean section) and their associated costs. These will be collected during site visits to study hospitals. For analysis, a health economist will generate an Excel-based data report by analysing assembled cost data. The report will contain micro-activity costs for each component of the implementation as well as estimated unit costs for each mode of birth (which include the types and frequency of commodities used, staff time, and other ingredient costs identified by the team). If the trial shows benefit for the primary outcome, a cost–benefit analysis will be performed.

#### Training

Prior to commencement of the trial phase, site research staff will complete a training workshop on study procedures, according to the study Manual of Operations. This workshop emphasizes GCP standards, the need for accurate and thorough data reporting and vigilance in identifying, detecting and reporting any possible adverse events, safety concerns or protocol deviations. Country and hospital investigators will maintain a valid GCP certificate throughout the trial. Standardized trainings for relevant staff will also be conducted at study sites on how to use and interpret the LCG and Robson Classification. The WHO manual on how to use the Labour Care Guide will be used for provider training at participating hospitals. This will be augmented by a standardized training program and group exercises (including case studies), previously prepared by WHO.

## Discussion

Over 140 million women give birth each year worldwide and the proportion of births attended by skilled health personnel is steadily increasing [32]. The findings of this study will provide critical evidence on how the LCG—WHO’s new partograph that is intended for global use by skilled health personnel—can be most effectively implemented, and what (if any) benefits are associated with its implementation. As the first randomised trial using the LCG, this study will not only provide robust evidence to guide further LCG dissemination and implementation activities, but the package of education and training materials and tools used in the trial will be made publicly available for others to use.

This study has several strengths. It uses a data-driven, theory-informed approach to developing the LCG strategy, which will aim to address and leverage the factors known to affect partograph use in health facility settings. The LCG strategy includes several components that have been shown to individually reduce unnecessary CS use, such as promoting labour companionship, encouraging mobility during labour, and ensuring women have adequate pain relief, as well as consistent audit and feedback of CS use. The stepped-wedge design ensures that all participating hospitals will be implementing the LCG strategy at the conclusion of the trial, which we anticipate will be favourable to its long-term sustainability. The findings will also inform future LCG-related research, particularly with regards to effect size estimation and outcome selection.

We acknowledge that our primary hypothesis extends beyond feasibility only (as is typical of pilot studies) and will assess effectiveness of the intervention for CS use amongst women in Robson Group 1 [33]. However, the study has been described as a pilot trial with several reasons in mind. First, as a novel and complex intervention, it is not clear what the likely effect size will be for process of care outcomes such as CS and augmentation of labour. Second, while avoiding unnecessary obstetric intervention is itself an important goal, CS use is also an intermediate outcome. The effects of LCG implementation on other, often rarer, maternal and newborn health outcomes will probably remain unknown at the conclusion of the study due to lack of statistical power. We therefore anticipate that further, large-scale studies will be needed to establish the effects of introducing the LCG on important health utcomes such as several neonatal morbidity or mortality. Thirdly, embedded within the trial are several innovations for which we are exploring feasibility, including the measurement of women’s experiences through a postpartum survey that is tailored to intrapartum supportive care interventions, and the use of a “low-dose, high-frequency” approach to LCG education and training. The main findings and process evaluation might suggest that these approaches are not feasible or need to be modified. Finally, the setting in which this trial is being conducted may not be representative of (or similar to) other countries or health facilities, which might impact the adoption of LCG and its effects. In India, the LaQshya national initiative and hospital accreditation process [18] has a strong emphasis on respectful maternity care, which is well-aligned with WHO’s intrapartum care recommendations and foundational principles of the LCG. Contextual differences around obstetric intervention use (including baseline CS, amniotomy, augmentation, and episiotomy rates), as well as differences in the risk profile of obstetric populations, may also mediate effects of the LCG strategy. In particular, we deliberately sought hospitals with high rates of CS in order to explore if this could be safely reduced for women in Robson Group 1. We therefore anticipate that further trials using LCG in different settings and contexts—for example, in midwifery-led care settings, in facilities with low CS rates, or in countries where no national, women-centred intrapartum care policy framework is in place—will be required.

Results of this study will be published in peer-reviewed, open-access journals and widely disseminated through international networks and conferences. Currently, the LCG and a user’s manual are publicly available via the WHO website, though official WHO education and training materials have not yet been published.

## Supplementary Information

**Additional file 1:  Fig S1.** Trial diagram showing number of women with a gestational age >20 weeks by hospital and steps. **Table S1.** Women characteristics, by study period. **Table S2.** Effect of the intervention on cesarean section and other maternal process of care and health outcomes. **Table S3.** Effect of the intervention on maternal and perinatal health outcomes. **Table S4.** Effect of the intervention on women’s experience outcomes. **Table S5.** Serious adverse events by period. **Table S6.** Serious adverse events by the relation to the WHO LCG study at the intervention period. **Fig S2.** Rates of maternal death, neonatal death, stillbirth and other SAE by hospital and month.

## Data Availability

Not applicable, as the study data have not yet been completed. At completion of the study, data will be made available on reasonable request from the authors.

## Abbreviations

CIOMS: Council for International Organisation of Medical Science
COM-B: Capability, opportunity, motivation, behaviour
CONSORT: Consolidated Standards of Reporting Trials
CS: Caesarean section
DSMC: Data and Safety Monitoring Committee
GEE: Generalized estimating equation
ICC: Intra-cluster correlation
ID: Identification
IECS: Institute for Clinical Effectiveness and Health Policy
LCG: Labour Care Guide
LMIC: Low- and middle-income countries
SPIRIT: Standard Protocol Items: Recommendations for Interventional Trials
WHO: World Health Organization
WMA: World Medical Association
GCP: Good clinical practice
REDCap: Research electronic data capture
SAE: Serious adverse event
