# Supplementary material for: Implementing the WHO Labour Care Guide to reduce the use of Caesarean section in four hospitals in India: protocol and statistical analysis plan for a pragmatic, stepped-wedge, cluster-randomized pilot trial
Source: Reprod Health. 2023 Jan 20;20:18. doi: 10.1186/s12978-022-01525-4 (PMC9862839; doi:10.1186/s12978-022-01525-4)
Supplement: Supplementary file 1 — Additional file 1: Fig S1. Trial diagram showing number of women with a gestational age >20 weeks by hospital and steps. Table S1. Women characteristics, by study period. Table S2. Effect of the intervention on cesarean section and other maternal process of care and health outcomes. Table S3. Effect of the intervention on maternal and perinatal health outcomes. Table S4. Effect of the intervention on women’s experience outcomes. Table S5. Serious adverse events by period. Table S6. Serious adverse events by the relation to the WHO LCG study at the intervention period. Fig S2. Rates of maternal death, neonatal death, stillbirth and other SAE by hospital and month. [file 12978_2022_1525_MOESM1_ESM.docx]

**
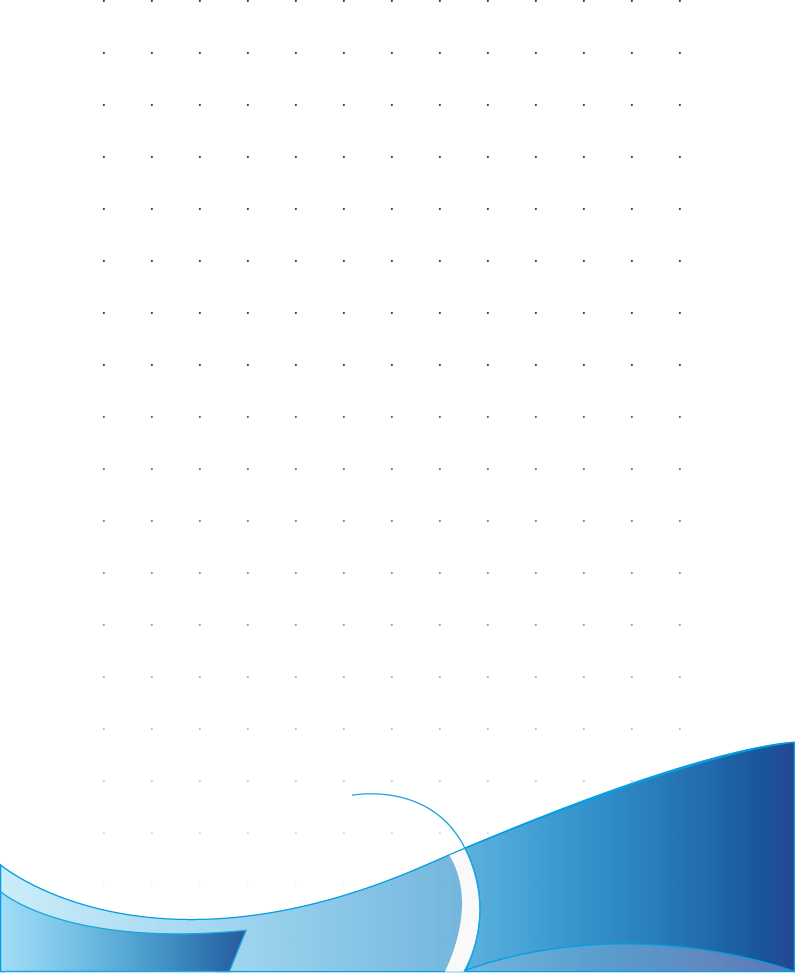

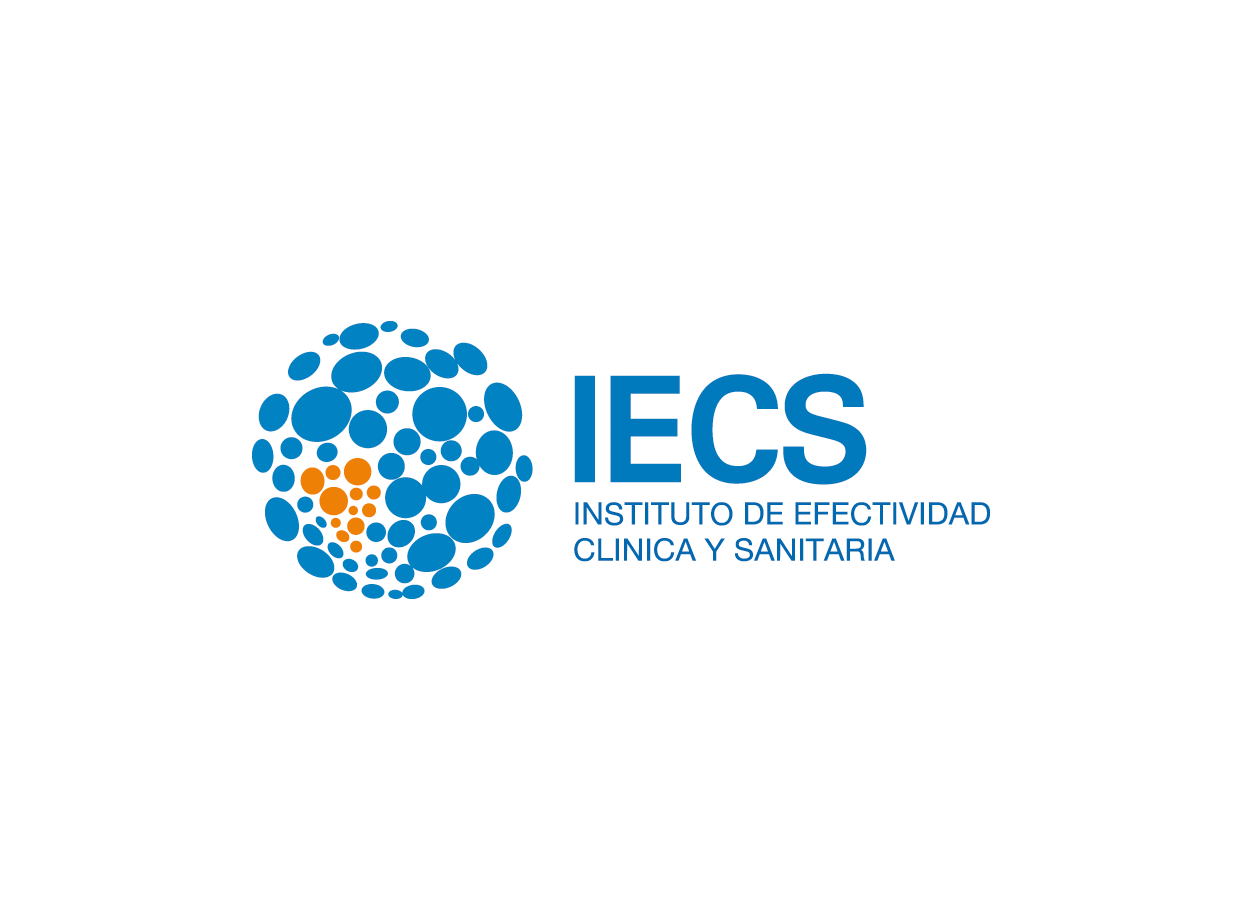
**

**Implementing the WHO Labour Care Guide to reduce the use of Caesarean section in four hospitals in India: a pragmatic, stepped wedge, cluster randomized pilot trial**

Statistical Analysis Plan

17 March 2022

Written by:

Biostatistics: L. Gibbons

Table of Contents

[**Section 1: Administrative information** 4](#_Toc99963735)

[Title and trial registration 4](#_Toc99963736)

[SAP Version 4](#_Toc99963737)

[Protocol Version 4](#_Toc99963738)

[Ethics approval 4](#_Toc99963739)

[SAP Revisions – revision history, with justification and timing 4](#_Toc99963740)

[Roles and Responsibility – non-signatory names and contribution 4](#_Toc99963741)

[Roles and Responsibility – signatures 4](#_Toc99963742)

[**Section 2: Introduction** 5](#_Toc99963743)

[Objectives, research hypotheses and study aims 5](#_Toc99963744)

[Study objectives 6](#_Toc99963745)

[**Section 3: Trial Methods** 6](#_Toc99963746)

[Trial design 6](#_Toc99963747)

[Sample Size 6](#_Toc99963748)

[Statistical Interim analyses 7](#_Toc99963749)

[**Section 4: Statistical Principles** 7](#_Toc99963750)

[Confidence intervals and p-values 7](#_Toc99963751)

[Protocol Deviations 7](#_Toc99963752)

[Analysis populations 7](#_Toc99963753)

[**Section 4: Trial Population** 7](#_Toc99963754)

[Screening Data – Coverage Data 7](#_Toc99963755)

[Eligibility 8](#_Toc99963756)

[Recruitment 8](#_Toc99963757)

[Loss to follow up 8](#_Toc99963758)

[End of trial definition 8](#_Toc99963759)

[Baseline patient characteristics 8](#_Toc99963760)

[**Section 5: Analysis** 8](#_Toc99963761)

[Primary Outcome 8](#_Toc99963762)

[Fetal/Neonatal Secondary Outcomes 10](#_Toc99963763)

[Women’s experience outcomes 11](#_Toc99963764)

[Women’s experience outcomes (cont.) 12](#_Toc99963765)

[Analysis methods 12](#_Toc99963766)

[Statistical Methods – adjustment for covariates 13](#_Toc99963767)

[Missing data 13](#_Toc99963768)

[Additional Analyses 13](#_Toc99963769)

[Statistical Software 13](#_Toc99963770)

[**Section 6. Dummy Tables** 14](#_Toc99963771)

[Figure 1: Trial diagram showing number of women with a gestational age >20 weeks by hospital and steps 14](#_Toc99963772)

[Table 1: Women characteristics, by study period 15](#_Toc99963773)

[Table 2: Effect of the intervention on cesarean section and other maternal process of care and health outcomes 16](#_Toc99963774)

[Table 3: Effect of the intervention on maternal and perinatal health outcomes 16](#_Toc99963775)

[Table 4: Effect of the intervention on women’s experience outcomes 17](#_Toc99963776)

[Table 5: Serious adverse events by period 18](#_Toc99963777)

[Table 6: Serious adverse events by the relation to the WHO LCG study at the intervention period 18](#_Toc99963778)

[Figure 2: Rates of maternal death, neonatal death, stillbirth and other SAE by hospital and month 18](#_Toc99963779)

# **Section 1: Administrative information**

# Title and trial registration

Statistical analysis plan for the LCG Study: a pragmatic, stepped wedge, cluster randomized pilot trial to reduce the use of Caesarean section in four hospitals in India implementing the WHO Labour Care Guide.

Trial protocol is registered with the Clinical Trials Registry of India (CTRI/2021/01/030695).

# SAP Version

Version: 2.0. Date: 17 March 2022

# Protocol Version

This document has been written based on information contained in the study protocol version 1.4, dated 17 March 2022

# Ethics approval

The trial protocol was reviewed and approved by the Alfred Hospital Human Ethics Committee (737/20), the Institutional Ethics Committee of the KLE Academy of Higher Education and Research, Belagavi, Karnataka, India (KAHER/EC/2020-21/D-281120003); the Institutional Ethics Committee of J J M Medical College, Davanagere, Karnataka (JJMMC/IEC- 136/2020); the Institutional Ethics Committee of Vijayanagar Institute of Medical Sciences (VIMS), Ballari, Karnataka (VIMS/STD/SVN IEC/20/2020-2021), the Institutional Ethics Committee, Gadag Institute of Medical Sciences, Gadag, Karnataka (GIMS/IEC/01/2020-21), the State Ethics Committee, Department of Health and Family Welfare, Government of Karnataka (DD(MH)/71/2020-21); and the Health Ministry's Screening Committee, Indian Council of Medical Research (2020-10127)

# SAP Revisions – revision history, with justification and timing

| **Version** | **Date** | **Justification** |
| --- | --- | --- |
| Version 1.0 | 22/09/2021 |  |
| Version 2.0 | 17/03/2022 | Changes in the methodology to be used for the main outcome was done. |

# Roles and Responsibility – non-signatory names and contribution

Luz Gibbons, Institute of Clinical Effectiveness and Health Policy. Senior Statistician.

# Roles and Responsibility – signatures

Signature Luz Gibbons:

Signature Joshua Vogel:

Signature Shivaprasad Goudar:

# **Section 2: Introduction**

# Objectives, research hypotheses and study aims

The study hypothesis is that the adoption of the LCG strategy, including use of LCG as a labour monitoring-to-decision tool, will enhance the quality of care during labour and birth, reduce use of unnecessary interventions and improve support to women during labour. This can reduce unnecessary intrapartum CS use.

The LCG has been designed for the care of women and their babies during labour and birth. It includes assessments and observations that are essential for the care of all pregnant women, regardless of their risk status. The LCG was primarily designed to be used for the care of apparently healthy pregnant women and their babies (i.e. women with low risk pregnancies). Women at high risk of developing labour complications may require additional monitoring and interventions, based on their particular clinical situation.

While this trial will measure the impacts of LCG implementation in the overall population of women giving birth, our hypothesis includes the following considerations:

- Women with nulliparous, singleton, cephalic, term pregnancies who experience spontaneous labour (ie. women in Robson Group 1) are women in whom overuse of CS is a recognised problem. For example, a multi-country analysis (287 hospitals, 21 countries) found that CS rates often exceeded 20% in these women.(10) However, WHO advises that CS rates of under 10% are achievable in this group of women, with good perinatal health outcomes.(11)
- Women who are admitted for antepartum CS do not experience labour, and therefore do not require a LCG (or any partograph) as part of their care. We therefore do not expect antepartum CS use to be affected.
- Some women with certain conditions or complications (such as women with a multiple pregnancy, a breech pregnancy, or a fetus in a transverse or oblique lie) account for a small fraction of the population, but have a high likelihood of requiring a CS. For example, ~2% of women would have a multiple pregnancy, in whom the CS rate is normally around 60%.(11) Women with a fetus in a transverse or oblique lie account for <1% of all women giving birth, but the CS rate is 100% in these women as vaginal birth is not possible. (11) The LCG would not be expected to reduce CS in such subgroups of women.

We therefore hypothesise that the main impact of the LCG implementation strategy will be detected amongst women with nulliparous, singleton, cephalic, term pregnancies who experience spontaneous labour (ie. women in Robson Group 1).

The study aims are:

1. Develop and optimise a strategy for implementing the LCG *(formative phase)*
2. To evaluate the implementation of the LCG strategy compared with usual care *(trial phase)*

# Study objectives

The primary objective is to evaluate the effect of operationalisation of the LCG implementation strategy on CS rate amongst women in Robson Group 1.

Secondary objectives are:

- Evaluate the effect of the LCG implementation strategy on women’s health and process of care outcomes and women’s experiences of care
- Conduct a process evaluation on the implementation of the LCG strategy

# **Section 3: Trial Methods**

# Trial design

The study is a pragmatic, stepped-wedge, cluster-randomized pilot trial. The step in which each hospital will start the intervention is randomized.

# Sample Size

This pilot trial will use a health outcome (CS rate in Robson Group 1) to evaluate the effects of the LCG implementation strategy. However, as this is a new complex intervention, the estimated effect size and ICC is difficult to estimate. One important output of this pilot trial will be to better estimate these measures for future, larger trials.

The four hospitals in total have on average approximately 24,000 births per year (around 4,000 births every 2 months) and the overall CS rate across all hospitals is approximately 44%. We estimate the current CS rate in women in Robson Group 1 in these four hospitals to be at least 40%. Across all four hospitals, approximately 1300 women in Robson Group 1 give birth every 2 months (i.e. an average of 325 women per cluster).

The trial will use 4 steps, 1 cluster in each step, a CAC of 90% and a cluster size of 300 women per step.

**Power estimation considering 4 steps, 1 cluster in each step, a CAC of 90% and a cluster size of 300 women (Coefficient of variation of cluster size 0.60)**

| **Baseline CS Rate in Robson Group 1** | **ICC** | **Relative Reduction** | | | |
| --- | --- | --- | --- | --- | --- |
|  |  | **35%** | **30%** | **25%** | **20%** |
| **40%*** | **0.01** | 100% | 99% | 97% | 86% |
|  | **0.02** | 100% | 99% | 92% | 76% |
|  | **0.05** | 96% | 88% | 74% | 54% |
| **30%**** | **0.01** | 99% | 96% | 88% | 70% |
|  | **0.02** | 97% | 91% | 78% | 58% |
|  | **0.05** | 85% | 73% | 56% | 38% |

*CS at the intervention period: 26%, 28%, 30%, 32%

**CS at the intervention period: 19.5%, 21%, 22.5%, 24%

The trial will provide 92% power to detect a 25% reduction in the Robson Group 1 CS rate from 40% to 30% (ICC 0.02). Should the ICC be higher than expected (eg: 0.05), or the CS rate in Robson Group 1 is lower than expected (eg: 30%) we will have >80% power to detect a 30% relative reduction (from 40% to 28%).

A separate power estimation was also perform for 240 women per cluster per step. The trial will provide 87% power to detect a 25% reduction in the Robson Group 1 CS rate from 40% to 30%.

# Statistical Interim analyses

One formal statistical interim analysis is planned to monitor adverse events outcomes and safety outcomes. This interim analysis is planned to take place in January 2022 and will be presented to the DSMC members. Every outcome in table 3 and table 5 will be reported by hospital and by month. Table 6 will also be included.

# **Section 4: Statistical Principles**

# Confidence intervals and p-values

All applicable statistical tests will be 2-sided and will be performed using a 5% significance level and all confidence intervals presented will be 95% and two-sided.

# Protocol Deviations

The number and type of protocol deviations will be reported by study period.

# Analysis populations

The intention-to-treat population will include all women who had a delivery in the randomised hospitals with a gestational age equal or higher 20 weeks.

A smaller population will be considered to answer the women’s experience secondary outcomes. The population for these outcomes will be women in Robson groups 1 and 3 aged 18 years or more with a baby alive, able to complete the survey and who provided written consent. Surveys were conducted during 15*-day* periods every 2 months during the trial phase.

No per-protocol population is considered.

# **Section 4: Trial Population**

# Screening Data – Coverage Data

The number of births with a gestational age equal or higher than 20 weeks, the number of babies born alive, dead and with vital status unknown are reported daily by the sites using a Coverage form. It is expected that the number of Birth forms for women with a gestational age equal or higher 20 weeks and the status of the babies born to those women are the same as those reported in the Coverage form. The percentage of the coverage will be calculated as: number of births recorded in Birth forms/total number births reported in the Coverage form. This summary will be provided overall and by hospitals

# Eligibility

The number of ineligible women (or births), if any, will be reported, with reasons for ineligibility (for example, women with gestational age less than 20 weeks). This will be done also for the survey data.

# Recruitment

A Trial diagram will report the number of total births and the total number of women surveyed by month and by hospitals.

# Loss to follow up

Loss to follow up is defined as the number of women/babies in whom the vital status at day 7 or discharge is not known. The numbers of women and infants that were lost to follow-up over the course of the trial will be reported.

# End of trial definition

The end of trial is defined as the final data capture from all participating sites, all births up to and including the date 31 July 2022 will be included.

# Baseline patient characteristics

Women will be described with respect to age, gravida, parity, previous CS, if they receive antenatal care during pregnancy, their COVID status at admission, if they were transferred from another health facility during labour, gestational age at delivery by the two periods: control and intervention.

Categorical data will be summarised by numbers and percentages. Continuous data will be summarised by mean and SD. Tests of statistical significance will not be routinely undertaken for baseline characteristics; rather the clinical importance of any imbalance will be noted.

# **Section 5: Analysis**

# Primary Outcome

CS rate amongst women in Robson Group 1 (i.e. women who are nulliparous, singleton, cephalic, ≥37 weeks’ gestation, in spontaneous labour). The numerator will be women in Robson Group 1 who had a CS and the denominator the number of women in Robson group 1.

Maternal Secondary Outcomes

| **Outcome** | **Outcome definition** |
| --- | --- |
| CS rate in women in Robson Groups 1 and 3 | Numerator: Number of women undergoing CS  Denominator: Number of women in Robson Groups 1 and 3 |
| CS rate in women in Robson Groups 1 to 5 | Numerator: Number of women undergoing CS  Denominator: Number of women in Robson Groups 1 to 5 |
| Overall CS rate | Numerator: Number of women undergoing CS  Denominator: Number of women giving birth |
| Augmentation with oxytocin during labour rate | Numerator: Number of women given oxytocin for augmentation during labour  Denominator: Number of women who experienced spontaneous labour |
| Artificial rupture of the membranes rate | Numerator: Number of women who had artificial rupture of membranes  Denominator: Number of women who experienced spontaneous labour |
| Episiotomy rate | Numerator: Number of women who had episiotomy  Denominator: Number of women with vaginal birth |
| Operative vaginal birth rate | Numerator: Number of women who had operative vaginal birth (forceps or vacuum)  Denominator: Number of women with vaginal birth |
| Duration of hospital admission | Total length (hours) of hospital admission for childbirth |
| 3^rd^ or 4^th^ degree tears | Numerator: Number of women experiencing 3^rd^ or 4^th^ degree tears  Denominator: Number of women giving birth |
| PPH requiring uterine balloon tamponade or surgical intervention | Numerator: Number of women requiring uterine balloon tamponade OR surgical intervention for PPH  Denominator: Number of women giving birth |
| Suspected or confirmed maternal infection requiring therapeutic antibiotics | Numerator: Number of women with clinical signs or symptoms of maternal infection AND therapeutic antibiotics were required  Denominator: Number of women giving birth |

# Fetal/Neonatal Secondary Outcomes

| **Outcome** | **Outcome definition** |
| --- | --- |
| Antepartum stillbirth | Numerator: Fetal death prior to admission  Denominator: All born babies |
| Intrapartum stillbirth | Numerator: Fetal death after admission  Denominator: All born babies |
| Apgar score <7 at 5 minutes | Numerator: Liveborn babies with Apgar <7 at 5 minutes  Denominator: Liveborn babies |
| Bag and mask ventilation of newborn | Numerator: Use of continuous bag and mask ventilation of newborn for >1 minute  Denominator: Liveborn babies |
| Mechanical ventilation of newborn | Numerator: Use of mechanical ventilation of newborn  Denominator: Liveborn babies |
| Prolonged (>48 hour) admission in NICU | Numerator: Admission to NICU for >48 hours  Denominator: Liveborn babies |
| Newborns requiring NICU admission for hypoxic ischaemic encephalopathy | Numerator: Admission to NICU for suspected or confirmed  Denominator: Liveborn babies |
| Neonatal death | Numerator: Neonatal death in a liveborn infant by day 7 or discharge (whichever came first)  Denominator: All liveborn babies |

# Women’s experience outcomes

| **Outcome** | **Outcome definition** |
| --- | --- |
| Woman’s experience with labour companion | Numerator: Women who reported a labour companion was present during labour or birth  Denominator: Women in Robson Group 1 or 3 who completed the survey |
| Woman’s experience of being offered pain relief | Numerator: Women who reported that they were asked whether they would like any pain relief  Denominator: Women in Robson Group 1 or 3 who completed the survey |
| Women’s satisfaction with their pain management during labour and birth | Numerator: Women who reported being very satisfied or somewhat satisfied with how their pain was managed during labour and birth  Denominator: Women in Robson Group 1 or 3 who completed the survey |
| Woman’s experience of being encouraged to drink oral fluids | Numerator: Women who reported that a health worker encouraged them to drink water  Denominator: Women in Robson Group 1 or 3 who completed the survey |
| Woman’s experience of being encouraged to eat food | Numerator: Women who reported that a health worker encouraged them to eat food  Denominator: Women in Robson Group 1 or 3 who completed the survey |
| Woman’s experience of mobilising during labour | Numerator: Women who reported that a health worker encouraged them to walk around during labour  Denominator: Women in Robson Group 1 or 3 who completed the survey |
| Woman’s experience of birth position of choice | Numerator: Women who reported that a health worker asked them which birth position they preferred  Denominator: Women in Robson Group 1 or 3 who completed the survey |
| Woman’s experience of time health worker spent with them | Numerator: Women who reported being very satisfied or somewhat satisfied with amount of time health worker spent with them during labour  Denominator: Women in Robson Group 1 or 3 who completed the survey |

# Women’s experience outcomes (cont.)

| **Outcome** | **Outcome definition** |
| --- | --- |
| Women’s satisfaction with the way health providers communicated with them | Numerator: Women who reported being very satisfied or somewhat satisfied with the way health workers communicated with them during labour and birth  Denominator: Women in Robson Group 1 or 3 who completed the survey |
| Woman’s experience of privacy | Numerator: Number of women who strongly agreed or agreed that their privacy was respected during examinations and treatments  Denominator: Women in Robson Group 1 or 3 who completed the survey |
| Women’s experience of being asked permission | Numerator: Number of women who said their health worker always asked permission before examinations and treatments  Denominator: Women in Robson Group 1 or 3 who completed the survey |
| Woman’s overall experience of care | Numerator: Number of women who strongly agreed or agreed that they felt satisfied with their labour and birth experience  Denominator: Women in Robson Group 1 or 3 who completed the survey |

# Analysis methods

All analyses will be by intention to treat. The primary comparison will be composed by the characteristics of the women enrolled at the control period versus those enrolled at the intervention period.

The primary comparison will be composed by the characteristics of the women enrolled at the control period versus those enrolled at the intervention period. For the primary outcome and secondary outcomes, a generalized estimating equation (GEE) will be used to estimate the effect of the intervention with respect to the population-average. An exchangeable correlation structure will be assumed and the binomial distribution with a log link function will be considered. The relative risk and the 95% confidence interval will be reported as the size effect. The model will be constructed considering two variables: a binary indicator for treatment – indicating whether the observation was made during the control or the intervention period and a categorical variable indicating the step (1-4) The selection of GEE model instead of mixed models was based on that GEE guarantees consistent estimators for the regression parameters even when the working correlation is misspecified (1).

These estimates can be biased when the number of clusters is small as it is in the LCG trial. There are different methods of bias correction and degree of freedom (df) approximation that can be apply in GEE models to maintain the validity of the estimations (2). Manck and DeRouen correction with N-2 degree of freedom will be used as it is the most conservative option (3).

The same methodology will be used for the outcome “Duration of hospital admission in days” changing the distribution into a Poisson as the possible values goes from 0 to 7+2.

# Statistical Methods – adjustment for covariates

Due to the pandemic situation, it is possible that an adjusted analysis by the COVID status of the women at admission will be conducted. The variable will be included in the model as a fixed effect variable.

It will be analyzed if the percentage of women in group 1 remains constant thought the trial. In the context of the pandemic, it is possible that the population attending the hospital varies in relation to risk or other factors. If the percentage of women in Robson group 1 changes through the duration of the trial, an adjustment by this percentage will be done.

It will also be evaluated if it necessary to adjust by the percentage of COVID at the hospital level or at the country level. The behavior of the woman or providers could be affected by the pandemic situation.

# Missing data

No missing data is expected in the primary outcome. If missing data is presented, no imputation will be done and those cases will not be considered in the analysis.

# Additional Analyses

Serious adverse events (maternal deaths, neonatal deaths, stillbirths and other) will be described across hospitals by month. The number and proportion of the global maternal deaths, neonatal deaths and stillbirths stratify by the gestational age at delivery will be reported by study period. No formal statistical testing will be undertaken for SAEs (though it is noted that stillbirth and neonatal death are included in the list of secondary outcomes, and will be analyzed as described above).

For every type of serious adverse events, it will be reported if the adverse event appears to be related, possible or probable related and not related with the LCG study during the intervention period.

# Statistical Software

The analysis will be carried out using R version 4.1.1.

##

# **Section 6. Dummy Tables**

# Figure 1: Trial diagram showing number of women with a gestational age >20 weeks by hospital and steps

**Month**

| Hospital | 1 | 2 | 3 | 4 | 5 | 6 | 7 | 8 | 9 | 10 | 11 | 12 | 13 | Total (N) |
| --- | --- | --- | --- | --- | --- | --- | --- | --- | --- | --- | --- | --- | --- | --- |
| 1 |  |  |  |  |  |  |  |  |  |  |  |  |  |  |
| 2 |  |  |  |  |  |  |  |  |  |  |  |  |  |  |
| 3 |  |  |  |  |  |  |  |  |  |  |  |  |  |  |
| 4 |  |  |  |  |  |  |  |  |  |  |  |  |  |  |
| Total (N) |  |  |  |  |  |  |  |  |  |  |  |  |  |  |

|  | Control Study period |  | Intervention study period |
| --- | --- | --- | --- |

# Table 1: Women characteristics, by study period

| **Variables** | **Control period**  **(N=)** | | **Intervention period (N=)** | |
| --- | --- | --- | --- | --- |
| **Socio-demographic characteristics** | **n/N** | **%** | **n/N** | **%** |
| Age in years* |  |  |  |  |
| Age by categories |  |  |  |  |
| - Less than 20 |  |  |  |  |
| - 20-34 |  |  |  |  |
| - 35 o more |  |  |  |  |
| **Obstetric History** |  |  |  |  |
| **Previous Cesarean Section** |  |  |  |  |
| - None |  |  |  |  |
| - 1-3 |  |  |  |  |
| Gravida |  |  |  |  |
| - None |  |  |  |  |
| - 1-3 |  |  |  |  |
| - 4 or more |  |  |  |  |
| Parity |  |  |  |  |
| - None |  |  |  |  |
| - 1-3 |  |  |  |  |
| - 4 or more |  |  |  |  |
| **Current pregnancy** |  |  |  |  |
| Women receive antenatal care during pregnancy |  |  |  |  |
| Covid status at admission |  |  |  |  |
| Transferred from another HF during labour |  |  |  |  |
| Gestational age at delivery* |  |  |  |  |

*Media and Standard deviation

|  | **Control period**  **(N=)** | **Intervention period (N=)** | **Relative Risk (95% CI)** | **P-value** |
| --- | --- | --- | --- | --- |
|  | **n/N %** | **n/N %** |  |  |
| **Primary outcome** |  |  |  |  |
| Cesarean section in Robson Group 1 |  |  |  |  |
| **Maternal Process Health Outcomes** |  |  |  |  |
| Cesarean section in women in Robson Groups 1 and 3 |  |  |  |  |
| Cesarean section in women in Robson Groups 1 to 5 |  |  |  |  |
| Overall cesarean section |  |  |  |  |
| Augmentation with oxytocin during labour |  |  |  |  |
| Artificial rupture of the membranes |  |  |  |  |
| Episiotomy |  |  |  |  |
| Operative vaginal birth |  |  |  |  |
| Duration of hospital admission* |  |  |  |  |

# Table 2: Effect of the intervention on cesarean section and other maternal process of care and health outcomes

# Table 3: Effect of the intervention on maternal and perinatal health outcomes

|  | **Control period**  **(N=)** | **Intervention period (N=)** | **Relative Risk (95% CI)** | **P-value** |
| --- | --- | --- | --- | --- |
|  | **n/N %** | **n/N %** |  |  |
| **Maternal Secondary Outcomes** |  |  |  |  |
| 3rd or 4th degree tears |  |  |  |  |
| PPH requiring uterine balloon tamponade or surgical intervention |  |  |  |  |
| Suspected or confirmed maternal infection requiring therapeutic antibiotics |  |  |  |  |
| **Fetal/Neonatal Secondary Outcomes** |  |  |  |  |
| Antepartum stillbirth |  |  |  |  |
| Intrapartum stillbirth |  |  |  |  |
| Apgar score <7 at 5 minutes |  |  |  |  |
| Bag and mask ventilation of newborn |  |  |  |  |
| Mechanical ventilation of newborn |  |  |  |  |
| Prolonged (>48 hour) admission in NICU |  |  |  |  |
| Newborns requiring NICU admission for hypoxic ischaemic encephalopathy |  |  |  |  |
| Neonatal death |  |  |  |  |

# Table 4: Effect of the intervention on women’s experience outcomes

|  | **Control period**  **(N=)** | **Intervention period (N=)** | **Relative Risk (95% CI)** | **P-value** |
| --- | --- | --- | --- | --- |
|  | **n/N %** | **n/N %** |  |  |
| **Women’s experience outcomes** |  |  |  |  |
| Woman reporting labour companion |  |  |  |  |
| Women reporting being offered pain relief |  |  |  |  |
| Women reporting being very satisfied or somewhat satisfied with how their pain was managed |  |  |  |  |
| Women reporting being encouraged to drink water |  |  |  |  |
| Women reporting being encouraged to eat food |  |  |  |  |
| Women reporting being encouraged to walk |  |  |  |  |
| Women reporting being asked which birth position they preferred |  |  |  |  |
| Women reporting being very satisfied or somewhat satisfied with the amount of time health provider spent with them |  |  |  |  |
| Women reporting being very satisfied or somewhat satisfied with the way health provider communicated with them |  |  |  |  |
| Women reporting being strongly agreed or agreed that their privacy was respected |  |  |  |  |
| Women reporting being asked permission before examinations and treatments |  |  |  |  |
| Women reporting being strongly agreed or agreed with satisfaction with their labour and birth experience |  |  |  |  |

# Table 5: Serious adverse events by period

|  | **Control period**  **(N=)** | **Intervention period**  **(N=)** |
| --- | --- | --- |
|  | **n/N %** | **n/N %** |
| Maternal Death |  |  |
| Neonatal death by GA at delivery |  |  |
| - 20 -28 weeks |  |  |
| - 28 weeks or more |  |  |
| Stillbirth by GA at delivery |  |  |
| - 20 -28 weeks |  |  |
| - 28 weeks or more |  |  |

# Table 6: Serious adverse events by the relation to the WHO LCG study at the intervention period

|  | **Intervention period**  **(N=)** |
| --- | --- |
|  | **n/N %** |
| Maternal Death |  |
| - Related |  |
| - Possible/Probable |  |
| - Not related |  |
| Neonatal death |  |
| - Related |  |
| - Possible/Probable |  |
| - Not related |  |
| Stillbirth |  |
| - Related |  |
| - Possible/Probable |  |
| - Not related |  |
| Other |  |
| - Related |  |
| - Possible/Probable |  |
| - Not related |  |

# Figure 2: Rates of maternal death, neonatal death, stillbirth and other SAE by hospital and month

1. Hubbard AE, Ahern J, Fleischer NL, Van der Laan M, Lippman SA, Jewell N, Bruckner T, Satariano WA. To GEE or not to GEE: comparing population average and mixed models for estimating the associations between neighborhood risk factors and health. Epidemiology. 2010 Jul;21(4):467-74. doi: 10.1097/EDE.0b013e3181caeb90. PMID: 20220526.
2. Ford WP, Westgate PM. Maintaining the validity of inference in small-sample stepped wedge cluster randomized trials with binary outcomes when using generalized estimating equations. Stat Med. 2020 Sep 20;39(21):2779-2792. doi: 10.1002/sim.8575. Epub 2020 Jun 23. PMID: 32578264.
3. Mancl LA, DeRouen TA. A covariance estimator for GEE with improved small-sample properties. Biometrics. 2001 Mar;57(1):126-34. doi: 10.1111/j.0006-341x.2001.00126.x. PMID: 11252587
